# Efficacy and mechanism of *Baicao Fuyanqing suppository* on mixed vaginitis based on 16S rRNA and metabolomics

**DOI:** 10.3389/fcimb.2023.1166366

**Published:** 2023-09-14

**Authors:** Qi Wang, Pengjiao Wang, Minyan Yuan, Min Zhang, Shuo Zhang, Xiaodong Sun, Leyuan Shang, Yujie Liu, Yanni Zhao, Nan Jiang, Xiuli Gao

**Affiliations:** ^1^ State Key Laboratory of Functions and Applications of Medicinal Plants and School of Pharmacy, Guizhou Medical University, Guiyang, China; ^2^ Center of Microbiology and Biochemical Pharmaceutical Engineering, Guizhou Medical University, Guiyang, China; ^3^ Experimental Animal Center, Guizhou Medical University, Guiyang, China; ^4^ Research and Development Department, Changsheng Pharmaceutical Co. Ltd., Guizhou, China

**Keywords:** Chinese medicine suppository, mixed vaginitis, vaginal flora, Lactobacillus, lipid metabolism

## Abstract

**Background:**

Mixed vaginitis is the infection of the vagina by at least two different pathogens at the same time, both of which contribute to an abnormal vaginal environment leading to signs and symptoms. *Baicao Fuyanqing suppository* (*BCFYQ*) is a Miao ethnomedicine, used to treat various vaginitis. The aim of this study was to investigate the efficacy and possible mechanism of *BCFYQ* in the treatment of mixed vaginitis based on 16S rRNA high-throughput sequencing and metabonomics.

**Methods:**

*Escherichia coli* and *Candida albicans* were used to establish mixed vaginitis model in SD rats. Three groups of low, medium and high doses (0.18/0.36/0.64 g.kg^-1^) were established, and administered vaginally once a day for 6 consecutive days. After the last administration, vaginal pH and IL-1β, IL-2, IL-13 and IgA levels were measured, and the vaginal tissue was examined pathologically. In addition, the vaginal flora was characterised by 16S rRNA, and endogenous metabolites in the vaginal tissue were detected by UHPLC-Q-Exactive MS.

**Results:**

Compared with the model group, *BCFYQ* can reduce the vaginal pH of rats, make it close to the normal group and improve the damaged vaginal epithelial tissue. The results of ELISA showed that *BCFYQ* decreased the levels of IL-1 β and IL-2 and increased the levels of IL-13 and IgA (*P*<0.05). In addition, *BCFYQ* may increase the abundance of vaginal flora, especially *Lactobacillus*. The differential metabolite enrichment pathway suggests that the therapeutic mechanism of *BCFYQ* is mainly related to lipid metabolism and amino acid metabolism.

**Conclusion:**

Our research shows that *BCFYQ* has a good therapeutic effect on mixed vaginitis. It repairs the damaged vaginal mucosa by regulating the vaginal flora and lipid metabolism disorders to improve the local immune function of the vagina and inhibit the growth and reproduction of pathogens.

## Introduction

Vaginitis is a common and diverse gynecological disease. It is a general term for a variety of vaginal mucosal inflammatory diseases caused by different pathogens invading the vagina. It is mainly divided into aerobic vaginosis (AV), bacterial vaginosis (BV), trichomonad vaginitis(TV) and Vulvovaginal Candidiasis (VVC). Mixed vaginitis is the infection of the vagina by at least two different pathogens at the same time, both of which contribute to an abnormal vaginal environment leading to signs and symptoms (Tumietto et al., 2019). Clinically more frequent mixed infection with bacteria and Candida albicans (Benyas and Sobel, 2022). Vaginitis, if not treated in time, leads to a decline in a woman’s local immune function and subsequent infection with other gynecological diseases such as cervicitis, pelvic inflammatory disease, cervical erosion, etc. Currently, antibiotics are the main treatment for vaginitis. However, they also kill the symbiotic bacteria, leading to an imbalance of the vaginal flora and also to antibiotic resistance of pathogens (Stojanović et al., 2012). Therefore, the search for effective alternative therapies with few side effects has become a new field of research.

The female vaginal microecology is one of the most important microecological systems in the human body, and it is directly or indirectly related to various gynecological diseases such as vaginitis. More and more researchers are advocating the treatment of vaginitis by improving the vaginal microecology. In recent years, probiotics used to combat vaginal dysbiosis have shown great promise in restoring normal vaginal flora, including preventing the loss of beneficial bacteria (Das et al., 2022). Therefore, in this study, vaginal microbial flora was used as a vital indicator, and high-throughput sequencing of 16S rRNA gene to investigate whether *BCFYQ* could treat vaginitis by improving vaginal microecology.

Traditional Chinese medicine suppositories for the treatment of vaginitis first appeared in the Western Han Dynasty in China. Studies have shown that traditional Chinese medicine suppositories have unique effects in the treatment of various gynecological inflammations, have fewer side effects, do not easily to cause drug resistance, and have advantages in restoring normal physiological functions of patients and eliminating potential causes (Ma et al., 2017). *BCFYQ* is a traditional Chinese medicine suppository developed from traditional Miao ethnomedicine (registration number: Z20026597). Its main ingredients are *Sophora flavescens* (0.64* g*), *Stemona japonica* (0.32* g*), *Cnidium monnieri* (L.) Cuss. (0.32 g), *Callicarpa macrophylla* (0.32* g*)*, Agrimonia Pilosa* (0.32* g*), Alum (0.01 g), Borneol (0.01 g), Camphor (0.06 g), and Boric acid (0.05 g). *Sophora flavescens* has been used for thousands of years to prevent and treat common ailments such as eczema, diarrhoea, vaginal itching and colitis (He et al., 2015). Studies have shown that the main active ingredient of *Stemona japonica* is alkaloid, which has the functions of anthelmintic, insecticidal, antitussive and anti-asthmatic, antitumor and antibacterial (But et al., 2012). Recent studies have shown that *Cnidium monnieri (L.) Cuss*. has a variety of pharmacological activities, mainly in anti-inflammatory, antioxidation and antibacterial itching (Tsai et al., 2015; Z. Wang and Shen, 2017). *Callicarpa macrophylla* has a variety of biological activities such as analgesic, anti-inflammatory, antipyretic, antifungal, antiarthritic, and antitumor (Chung et al., 2014; Feng et al., 2017; Z. H. Wang et al., 2019). *Agrimonia pilosa* has a long history of medicinal use and is also widely used for gynecological disorders (Y. M. Lee et al., 2012). TIAN Wen-yang’s review summarised the clinical studies on BCFYQ in the treatment of vaginitis, among which there were 2 literatures on the use of BCFYQ alone in the treatment of instructions for the indication of internal vaginitis, and they used case series observation and case control study methods respectively. The results showed that the drug alone was effective in treating vaginitis, and the efficacy was better than metronidazole suppository (Wen-yang, 2016). However, there is currently no research on the therapeutic effect of *BCFYQ* on mixed vaginitis, and its mechanism of action in the treatment of vaginitis has not yet been elucidated.

In this study, we investigate the therapeutic effect of *BCFYQ* on mixed vaginitis by modeling mixed vaginitis rats with *Escherichia coli* and *Candida albicans*. Finally, combined with tissue metabolomics and 16S rRNA high-throughput sequencing methods, we sought to determine its mechanism of action in the treatment of vaginitis.

## Materials and methods

### Medicines and reagents

*BCFYQ* (Guizhou Changsheng Pharmaceutical Co., Ltd., batch number: 06210401); Estradiol Benzoate Injection (Shanghai Quanyu Biotechnology Animal Pharmaceutical Co., Ltd., batch number: 210301).

### Preparation of mixed bacterial suspension

*Escherichia coli* (BNCC133264) and *Candida albicans* (BNCC186382) used in this study were purchased from Beina Chuanglian Biotechnology Co., Ltd., *Escherichia coli* was cultured in nutrient broth medium and placed in a 37°C incubator; *Candida albicans* was cultured in YM medium and placed in a 28°C incubator. Before use, were diluted to a concentration of 2~3×10^8^ CFU/mL, respectively, and then mixed to prepare a bacterial suspension (*Escherichia coli*: *Candida albicans*, 1:1).

### Animal experiment and grouping

Female Sprague-Dawley (SD) rats, weighing 180-200 g, SPF grade, were obtained from the Animal Center of Guizhou Medical University (No.2000350). Animal experiments were approved by the Animal Experiment Center of Guizhou Medical University and the Animal Ethics Committee of Guizhou Medical University, and were conducted according to the guidelines of the “National Laboratory Animal Welfare and Ethical Requirements for Animal Experiments” (License No. SYXK (Qian) 2018-0001). Briefly, 40 rats were housed in an animal room with a relative humidity of 60 ± 5% and a 12 h light/dark cycle at 22 ± 2°C. After 1 week of adaptive feeding, 32 SD female rats were randomly selected and injected subcutaneously with 0.1 mL of 2 mg/mL estradiol benzoate hormone for 6 days to induce false estrus. 32 SD female rats were injected daily for 6 days with 20 μL of the mixed bacterial suspension through the vaginal opening, and then randomly divided into the model group and three treatment groups (Low dose group: 0.184 g/kg; Medium dose group: 0.364 g/kg; High dose group: 0.64 g/kg). The treatment group was given a 6-days course, the Model group was given the same amount of normal saline, and the Normal group (no infection and no medication) was established. There were eight rats in each group.

Six days after treatment, the vaginal pH of all rats was measured using pH test paper (Hangzhou Shisan Technology Co., Ltd.).

### Sample collection

In a sterile environment, rat vaginal secretions were collected with sterile cotton swabs, immediately frozen in liquid nitrogen, and stored at -80°C.The vagina of each rat was lavaged with 0.5 mL of 0.9% sterile saline, and the vaginal lavage fluid was collected, immediately frozen with liquid nitrogen, and stored at -80°C.

After the experiment, the rats were sacrificed in a sterile environment, and the vaginal tissues were separated. Part of this tissue was immediately stored in liquid nitrogen and then at −80°C until further use for expression studies. The remainder of the tissue was fixed in buffered formalin (4%) for histopathological studies.

### Detection of cytokines

The frozen vaginal lavage fluid was placed at room temperature (24 ± 2°C) for 30 min. It was centrifuged at 1000 g for 10 minutes at 4°C. The supernatant was collected and set aside. According to the operating steps of the ELISA kit (Xinbosheng, Neobioscience, Shenzhen, China), the double antibody sandwich method was used to detect interleukin-1β (IL-1β), interleukin-2 (IL-2), interleukin-13 (IL-13) and secretory immunoglobulin A (sIgA) in vaginal lavage fluid.

### Histopathological studies

Vaginal tissues fixed in 4% formalin were collected, fixed tissues were embedded in paraffin, sectioned at 3-5 μm thickness, and stained with hematoxylin-eosin (H&E) for histopathological examination (Gilbert et al., 2013). Histological examination was performed under a light microscope (BX43, Olympus, Japan).

### Vaginal microflora analysis

Using OMEGAD kits (Omega Bio-Tek, Norcross, GA, USA), extract genomic DNA from cotton swab samples according to the manufacturer’s instructions. The purity and concentration of DNA were detected by Thermo NanoDrop One. Concentration and purity were measured using the NanoDrop One (Thermo Fisher Scientific, MA, USA). PCR amplification of the V3-V4 region of the bacterial 16S rRNA gene was performed using forward primer 338F (5′-ACTCCTACGGGAGGCAGCA-3′) and reverse primer 806R (5′-GGACTACHVGGGTWTCTAAT-3′). PCR products were mixed in equidensity ratios according to the GeneTools analysis Software (version4.03.05.0, SynGene). The mixture of PCR products was then purified using the E.Z.N.A. Gel Extraction Kit (Omega, USA). The library was sequenced on an Illumina Nova6000 platform and 250 bp paired-end reads were generated (Guangdong Magigene Biotechnology Co., Ltd., Guangzhou, China).

### Sample preparation and analysis for metabolomics

Based on the tissue sample processing method established by Velenosi et al. (Velenosi et al., 2016), we slightly modified it. Briefly, 100 mg of vaginal tissue was collected, 500 μL of water, acetonitrile and methanol (1:2:2) was added, vortexed and mixed for 5 min, ultrasonicated for 10 min, placed on ice for 30 minutes and centrifuge at 4°C for 15 minutes at 26775g.The supernatant was passed through a 0.22 μm filter. Samples were analyzed by UHPLC-Q-Exactive MS. The quality control (QC) sample used to assess the analytical variance of the data was mixed from each biological sample in the same volume.

### Data analysis

Normally distributed data are expressed as mean ± standard deviation (X ± SD). Data were analysed and visualised using SPSS 19.0 (IBM, Chicago, USA) and GraphPad Prism 8.0 (GraphPad Software, CA, USA). Statistical analysis was performed using one-way analysis of variance (ANOVA). P values less than 0.05 indicate statistical significance, where *P<0.05, **P<0.01. The differences between indices of alpha diversity analysis were analyzed by Kruskal-Wallis rank sum test. Use R to construct heat map and PCoA.

## Results

### Effect of BCFYQ on pH of rat vagina

After 6 days of administration, the vaginal pH values of the three administration groups were significantly different from that of the Model group, with statistical significance (**Table 1**, *P*<0.05), and returned to the level of the Normal group. This indicates that *BCFYQ* can restore the vaginal pH to normal level in rats with mixed vaginitis.

**Table 1:** Effects of *BCFYQ* on pH of Rats with mixed vaginitis.

Groups	Exposure	Day 6 intervention
Normal	—	6.93 ± 0.86
Model	—	7.87 ± 0.64^#^
Low-dose	0.18 g.kg^-1^	7.12 ± 0.69*
Middle-dose	0.36 g.kg^-1^	7.05 ± 0.79*
High-dose	0.64 g.kg^-1^	6.87 ± 0.69*

**Table 1** Data were analyzed using one-way analysis of variance (ANOVA). Results are expressed as mean x ± SD(N=8), compared with Normal group ^#^ *P*<0.05,compared with Model group *P<0.05.

### Histopathological studies

**Figure 1:**
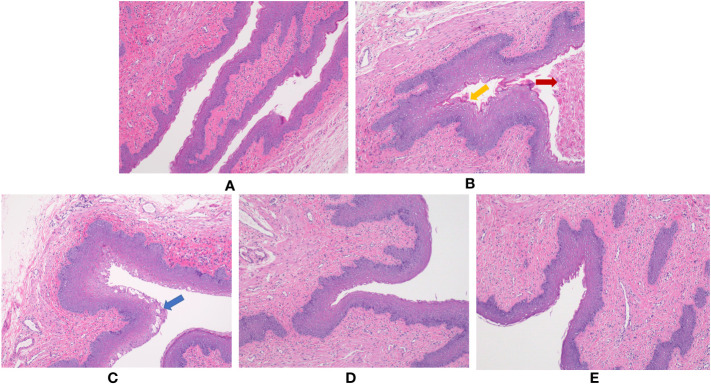
Effect of *BCFYQ* on vaginal tissue in rats with mixed vaginitis. **(A)** Normal group. **(B)** Model group. **(C)** Low-dose group. **(D)** Medium-dose group. **(E)** High-dose group. The red arrow indicates shedding of vaginal epithelial cells. The yellow arrow indicates edema and hyperplasia of vaginal epithelial tissue. The blue arrow indicates ulceration of vaginal epithelial tissue. All images were taken at 100x magnification.

Pathological studies of vaginal tissue were performed by H&E staining. The results showed that under the infection of *Escherichia coli* and *Candida albicans*, vaginal epithelial tissue showed edema and hyperplasia, a large number of epithelial cells were shed, and a large number of inflammatory cells were infiltrated. Compared with the Model group, the administration groups of the three doses had different degrees of improvement, and they approached the Normal group (**Figure 1**).

### Cytokine expression

**Figure 2:**
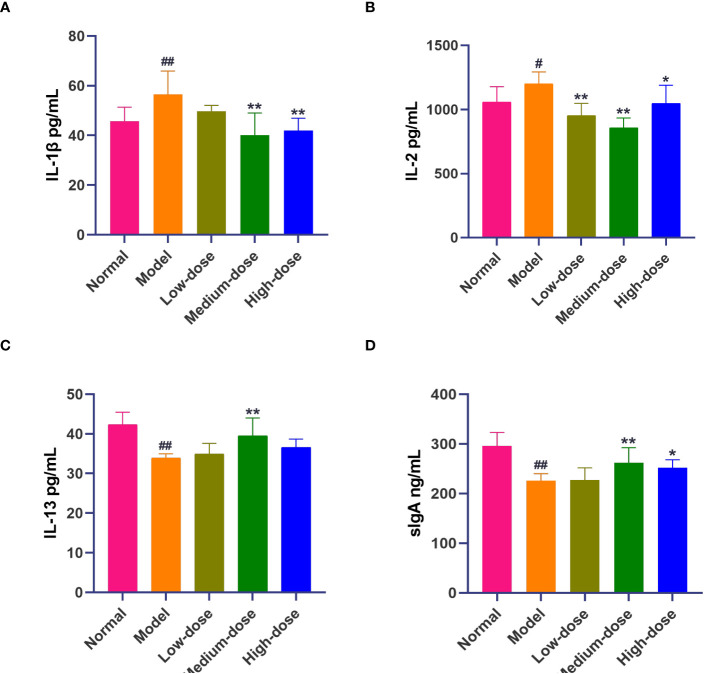
Effect of *BCFYQ* on cytokine levels in vaginal lavage fluid of rats with mixed vaginitis. **(A)** Differences in IL-1β levels. **(B)** Differences in IL-2 levels. **(C)** Differences in IL-13 levels. **(D)** Differences in sIgA levels. Each value is presented as the mean ± SD (n=8). Compared with the Normal group, ^#^ *P*<0.05, ^##^ *P*<0.01; compared with the Model group, *P<0.05, **P<0.01.

The results showed that the pro-inflammatory cytokines IL-1β and IL-2 in the Model group were significantly increased compared with those in the Normal group (56.48 ± 9.45 vs. 45.71 ± 5.68, *P*<0.01; 1200.45 ± 92.44 vs. 1057.59 ± 121.14, *P*<0.05). The levels of pro-inflammatory cytokines IL-1β and IL-2 were decreased in the BCFYQ-treated group compared to the Model group, with the effect being more pronounced in the Medium-dose group. (40.03 ± 9.00, *P <*0.01; 858.25 ± 76.00, *P <*0.01) (**Figures 2A, B**). The level of IL-13 in the Model group was significantly decreased compared with that in the Normal group (33.89 ± 1.08 vs. 42.35 ± 3.12, *P*<0.01), and the level of IL-13 in the treatment group was increased, and there was a significant difference between the Middle-dose group and the Model group (39.48 ± 4.55, *P*<0.01) (**Figure 2C**).

The level of secreted IgA (sIgA) in the Model group was significantly lower than that in the Normal group (226.28 ± 13.84 vs. 295.78 ± 27.3, *P*<0.05). In the treatment group, the Middle-dose group and the High-dose group were significantly higher than the Model group (262.29 ± 30.34, *P*<0.01; 252.21 ± 15.84, *P*<0.05) (**Figure 2D**).

### Vaginal microflora analysis

**Figure 3:**
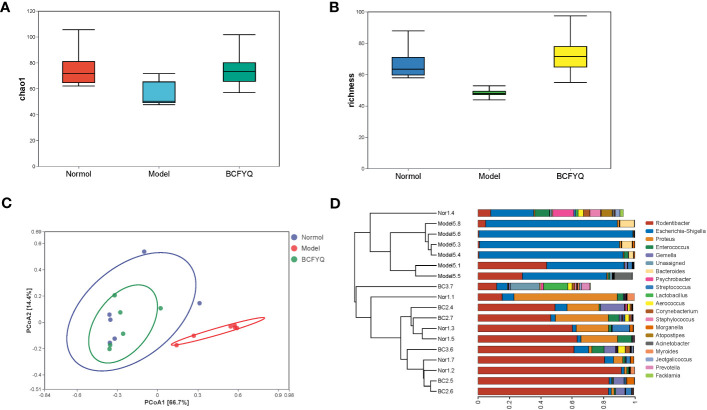
Analysis of alpha diversity. **(A)** The α-diversity indexes of Chao. **(B)** The α-diversity indexes of Richness. **(C)** Principal component analysis (PCoA) of vaginal flora microbiota based on Normal, Model and BCFYQ grouping. **(D)** Hierarchical clustering Analysis at Genus level.

Six samples were randomly selected from Normal, Model and BCFYQ groups for detection. To verify whether the amount of sequencing in this study was sufficient to reflect the diversity of the original microorganisms, the 16S rRNA sequencing results were analyzed for α-diversity (e.g., Chao1, Richness) based on the 97% similarity level. The Chao index reflects community richness, while Richness index reflects species richness. The statistical analysis of α diversity index showed that the community richness and species richness of the Normal group and the BCFYQ group were higher than those of the Model group (**Figures 3A, B**). Beta diversity refers to the comparison of biodiversity among different samples, which is represented by Principal Coordinate Analysis (PCoA) and hierarchical cluster analysis (**Figures 3C, D**). It can be seen that, the vaginal microbiota composition distribution between BCFYQ group and Model group is clearly separated, indicating that BCFYQ group can significantly improve the vaginal microbiota in mixed vaginitis rats. It is worth noting that the microbial composition distribution of the BCFYQ group and the Normal group almost overlapped, indicating that the BCFYQ group had a tendency to restore the disturbed vaginal flora of the mixed vaginitis rats to the normal level.

**Figure 4:**
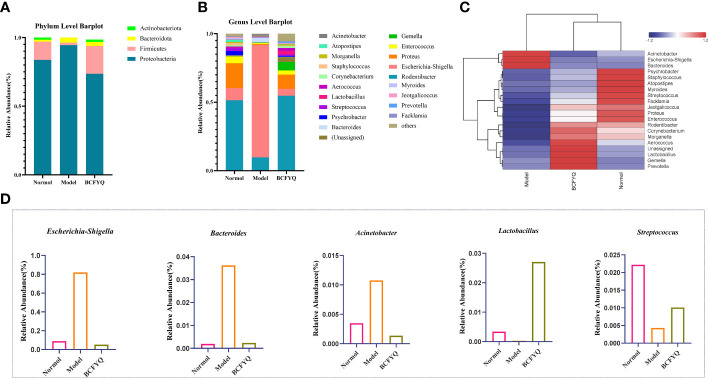
Analysis of beta diversity. **(A)** Relative percentages of community richness at the phylum level for the three groups. **(B)** Relative percentages of community richness at the genus level for the three groups. **(C)** Heat map analysis of communities at the genus level. **(D)** Comparison of relative abundance of Escherichia-Shigella, Bacteroides, Acinetobacter, Lactobacillus, and Streptococcus in the three groups.

**Figures 4A, B** show the results of phylum and genus level species annotation analysis for the three groups revealed by 16S rRNA sequencing. The results showed the top 4 most abundant bacteria at phylum level and the top 20 bacteria at genus level. At the phylum level, *Proteobacteria* and *Firmicutes* were the most important parts of the rat vaginal flora, accounting for the main relative abundance in all samples. Compared with the Model group, the *BCFYQ* group inhibited *Proteobacteria* and *Bacteroides*, and increased the abundance of *Firmicutes* and *Actinomycetes*. As can be seen from **Figure 4E**, compared with the Normal group, the *BCFYQ* group improved the Model group and restored the flora abundance to the level of the Normal group. We selected the top 20 genera at the genus level. The remaining species are grouped into Others, while Unassigned represents those species that are not classified and annotated. **Figure 4B** shows that *Escherichia-Shigella* was absolutely dominant in the Model group with a relative abundance of 0.8, while the sum of other bacteria only reached about 0.2, indicating that the vaginal flora of the rats in the Model group was disturbed by modelling. On the contrary, the *BCFYQ* group significantly reduced *Escherichia-Shigella*, *Bacteroides* and *Acinetobacter* compared to the Model group, but increased the relative abundance of *Lactobacillus* and *Streptococcus* (**Figure 4D**).

According to the species annotation and abundance information of all samples at the genus level, the top 20 genera in abundance were selected, and clustering was performed at the species and sample levels according to their abundance information in each group of samples. Plot as a heatmap to identify species that aggregate at higher or lower levels in the sample (**Figure 4C**). Our results showed that *Escherichia-Shigella*, *Acinetobacter* and *Bacteroides* were more abundant in the Model group than other groups, with Z>1. *Lactobacillus*, *Blautia*, *Coprococcus* and *Prevotella* were more abundant in *BCFYQ* group with Z>1. The abundance of *Psychrobacter*, *Staphylococcus*, and *Streptococcus* in the Normal group was higher than that in the other groups, Z>1. This is consistent with our previous observation that both the diversity and richness of the *BCFYQ* and Normal groups were better than those of the Model group. The genera with higher abundance in the Model group were all opportunistic pathogens. This also indicates that the Model group was successfully infected and disrupted its flora.

### Vaginal tissue metabolomic analysis

**Figure 5:**
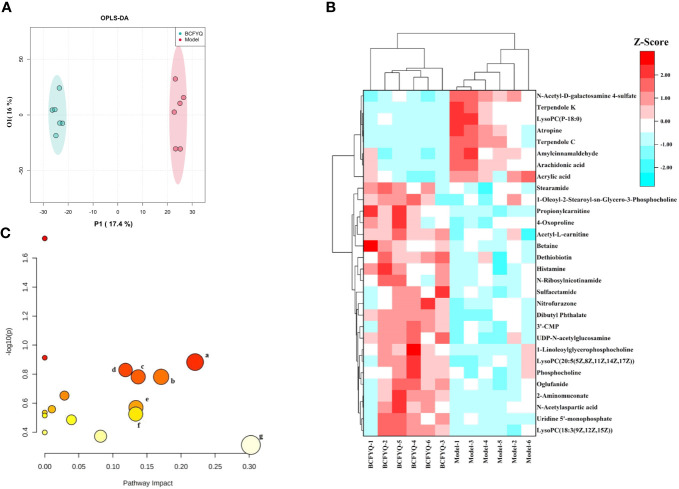
Effects of BCFYQ on vaginal metabolites in rats with mixed vaginitis. **(A)** OPLS-DA plots with the scores of the two principal components. **(B)** Heatmap of 30 differential metabolites. A standardized Z-score conversion was applied to convert concentration values to Z-scores prior to heatmap analysis. **(C)** Metabolic pathways of BCFYQ in the treatment of mixed vaginitis in rats (Impact value > 1). (A) Lactose Synthesis. (B) Phosphatidylcholine Biosynthesis. (C) Betaine Metabolism. (D) Alpha Linolenic Acid and Linoleic Acid Metabolism. (E) Amino Sugar Metabolism. (F) Histidine Metabolism. (G) Arachidonic Acid Metabolism.

As the vaginal flora changes, it will inevitably affect the endogenous metabolites of the vaginal tissue. We further explored the beneficial mechanism of *BCFYQ* by screening differential metabolites. Pairwise comparisons were established using the supervised mode OPLS-DA, and differences in metabolic profiles between the Model group and the *BCFYQ* group were observed. The OPLS-DA score plot showed that the *BCFYQ*-treated samples could be well separated from the Model group (**Figure 5A**). We created a heatmap by converting the concentration values ​​of the 30 differential metabolites to Z-scores by a normalized Z-score transformation (**Figure 5B**). The results showed that *BCFYQ* had a significant effect on vaginal tissue metabolism in mixed vaginitis. Among them, several metabolites such as acrylic acid, arachidonic acid, LysoPC (P-18:0), and amylcinnamaldehyde were significantly decreased after *BCFYQ* treatment. In addition, the treatment group increased levels of metabolites such as betaine, phosphocholine, dethiobiotin, and N-ribosylnicotinamide. We can preliminarily judge that the therapeutic effect of *BCFYQ* should be closely related to amino acid metabolism and lipid metabolism. After using Metaboanalyst to analyze the metabolic pathways of the above potential markers, the related pathways with influence value>0.1 were obtained according to the pathway topology analysis. The results showed that there were 7 significant pathways, which were related to several pathways including Lactose Synthesis, Phospholipid Biosynthesis, and Betaine Metabolism (**Figure 5C**).

### Correlation analysis of vaginal flora and metabolites

**Figure 6:**
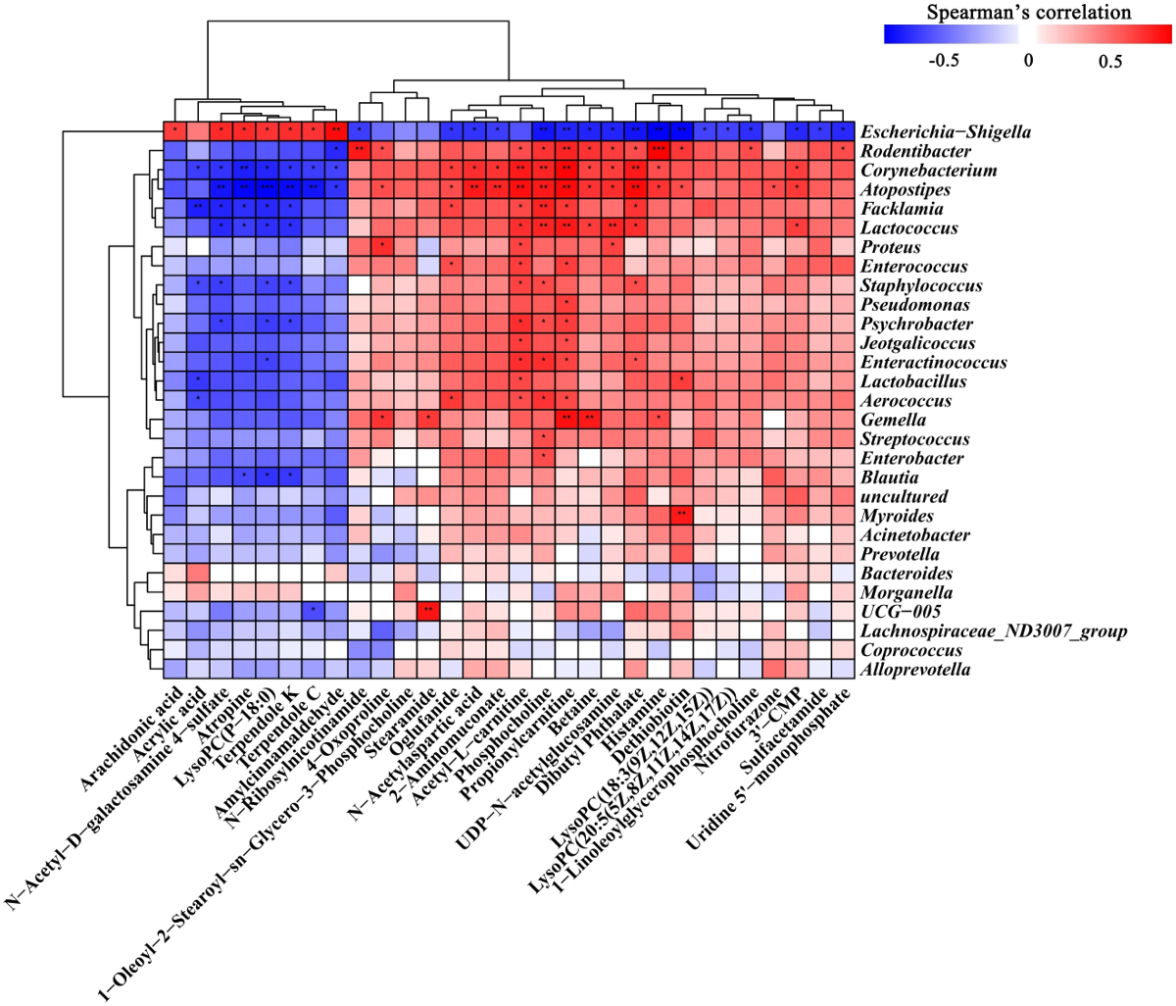
Spearman correlation between 29 altered vaginal microbiota and 30 metabolites. Red means positive correlation, blue means negative correlation. No identification means that the relevant confidence is less than 95%, which is not statistically significant. Sig < 0.05 is expressed by *, sig < 0.01 is expressed by **, and sig < 0.001 is expressed by ***, suggesting that there is a significant correlation between them.

We used Spearman rank correlation analysis to analyze the correlations between 30 differential metabolites and 29 altered vaginal microbiota (**Figure 6**). It can be seen from the figure that a variety of metabolites are significantly associated with vaginal flora. The results showed that *Lactobacillus* was negatively correlated with acrylic acid, and positively correlated with acetyl-L-carnitine and *Dethiobiotin*. *Escherichia-Shigella* was negatively correlated with *Phosphocholine*, *Propionylcarnitine* and *Dethiobiotin*, and positively correlated with amylcinnamaldehyde and arachidonic acid. We also observed that most of the metabolites were negatively correlated with *Escherichia-Shigella* and positively correlated with other vaginal flora. The results further suggest that *BCFYQ* alters vaginal endogenous metabolites by improving vaginal flora, thereby improving its inflammatory response or enhancing immune response.

## Discussion

According to a literature review evaluating the prevalence of mixed vaginitis (Rivers et al., 2011), the proportion of mixed vaginitis ranges from 4.44% to 35.06%. Currently, the mixed infection rates associated with AV are most notable among Chinese women. The mixed infection rates of AV+BV, AV+VVC and AV+TV are 36.9%, 38.1% and 25%, respectively (Fan et al., 2013). Therefore, the mixed vaginitis model used in this study is a mixed infection of *Escherichia coli* and *Candida albicans.* Estrogen is widely used to establish mouse models of bacterial infection. In addition to providing a high degree of stability, these models can stimulate successful colonization of the administered strains (Jerse et al., 2011). The pathogenesis of mixed vaginitis has not been fully elucidated. Some researchers have suggested that the pathogenesis of mixed vaginitis may be related to the mixed biofilm formed by bacteria and fungi (Beaussart et al., 2013). Lohse et al. (Lohse et al., 2018) proposed that the microbial populations in the host interact with each other in a synergistic or antagonistic form, so the pathogenesis of mixed vaginitis is also related to the imbalance of vaginal microbiota. Antibiotics are commonly used in clinical treatment of mixed vaginitis, but while killing pathogens, they will also kill beneficial bacteria, causing vaginal flora imbalance and leading to disease recurrence. Therefore, it has become a difficult problem to find drugs that are effective in treating vaginitis with less side effects. This study selected *BCFYQ*, a traditional Chinese medicine preparation, to explore its efficacy in the treatment of mixed vaginitis, and to make a basic exploration of its therapeutic mechanism.

*Sophora flavescens*, one of the main components of *BCFYQ*, has a strong inhibitory effect on bacteria and fungi by affecting the formation of biofilms, destroying cell walls, interfering in protein synthesis, and inhibiting bacterial division (Z. Li et al., 2022). *Sophora flavescens* is often prepared as a gel preparation alone to treat bacterial vaginosis, and it has been shown to have good anti-inflammatory activity (H. D. Zhao et al., 2016). In addition, *Stemona japonica*, *Cnidium monnieri (L.) Cuss*., *Callicarpa macrophylla* and *Agrimonia pilosa* have certain antibacterial and anti-inflammatory effects. It is worth noting that *BCFYQ* also contains boric acid. Some studies have reported that boric acid can destabilize the mycelial cytoskeleton and inhibit the invasive growth of *Candida albicans* (Pointer et al., 2015). Boric acid is commonly used as a pH regulator in industrial and pharmaceutical studies, so we infer that this is one of the reasons for lowering vaginal pH.

Studies have shown that the local immunity of the vagina plays a more important role than the systemic immunity, and its occurrence is related to local immune dysfunction (Budilovskaya et al., 2020). Th1/Th2 polarization is a key link in the regulation of immune response, and cytokines are the most important factors affecting the differentiation of Th cells. Th cells are mainly divided into Th1 and Th2. IL-2 is a pro-inflammatory factor secreted by Th1 cells, which can enhance the lethality of natural killer cells and mononuclear macrophages. When the cellular immune function of the body changes, the concentration of IL-2 will change. IL-13 is an anti-inflammatory molecule secreted by Th2 cells, which can stimulate the proliferation and differentiation of human B cells and secrete immunoglobulin, and plays an important role in humoral immunity. Our ELISA results showed that the secretion of IL-2 increased and the secretion of IL-13 decreased in rats infected with mixed bacteria. This suggests that mixed vaginitis may be related to Th1/Th2 immune imbalance. After *BCFYQ* treatment, the levels of IL-2 and IL-13 tended to return to normal levels. We can speculate that *BCFYQ* can reduce inflammation by inhibiting the secretion of proinflammatory factor IL-2 and promoting the secretion of anti-inflammatory factor IL-13, so as to re-balance Th1/Th2 immunity. IL-1β is one of the important inflammatory mediators, as well as a pyrogenic component, which plays a role in heating and mediating inflammation. It mainly plays a regulatory role in the activation of cellular immunity. Our results showed that *BCFYQ* did reduce the increase of IL-1β caused by infection, which was consistent with the results of Marconi et al. (Marconi et al., 2013).

Although some microorganisms, such as viruses and fungi, are controlled by cellular immunity, sIgA can also attach to the mucosa of the reproductive tract and play an important role in controlling these pathogens (Giraldo et al., 2006). If bacteria and extracellular parasites need to be removed, antibodies (immunoglobulins) are produced, constituting a humoral response (Brodsky, 1991). The results showed that sIgA secretion was significantly reduced in the infected rats compared with the Normal group, indicating that changes in vaginal mucosal tissue would impair IgA secretion, which would lead to changes in local immune function and a significant increase in this mucosal infection (Janković et al., 1995). After *BCFYQ* treatment, the content of sIgA tended to increase, and the middle dose returned to the normal level. This suggests that *BCFYQ* can enhance local humoral immunity in the vagina. In conclusion, *BCFYQ* plays an important role in anti-mixed bacterial vaginal infection through local cellular and humoral immunity.

The vaginal mucosa is the first line of defense for microbial contact, and vaginal epithelial cells increase immune responses by providing a physical barrier. The defense mechanism against this infection is demonstrated by the rapid shedding of epithelial cells, the presence of pattern recognition receptors, and the release of inflammatory cytokines (Balakrishnan et al., 2022). This was consistent with our H&E staining results. The Model group had a large number of vaginal epithelial cells shedding and accompanied by a large number of inflammatory cells invasion, while the three groups of rats after *BCFYQ* treatment had different degrees of improvement, among which the middle dose had the best effect. This indicates that *BCFYQ* has a healing effect on the damaged vaginal mucosa after mixed bacterial infection. *BCFYQ* restores immune function and reduces inflammation by repairing the vaginal mucosa.

The vaginal microbiota of healthy women of childbearing age is composed of aerobic and anaerobic bacteria, mainly *Lactobacillus* (Larsen and Monif, 2001). A large number of studies have shown that the disorder of vaginal microecology is one of the important factors leading to most gynecological diseases, among which different kinds of vaginitis are the most likely to be caused (Han et al., 2019). We characterized the vaginal microbiota of rats in the three groups by 16S rRNA high-throughput sequencing, and it could be seen that the community abundance and species richness of vaginal microbiota of rats in the Model group was reduced. Among them, pathogenic bacteria such as *Escherichia-Shigella* were the dominant bacteria, which occupied a dominant position in the whole vaginal microbiota, while beneficial bacteria such as *Lactobacillus* significantly decreased. This shows that our model is successful. Our results showed that the vaginal microbiota of rats treated with *BCFYQ* returned to a normal state, and the richness of microbiota at both phylum and genus levels was significantly increased. What is more, treatment with *BCFYQ* can promote the growth and reproduction of *Lactobacillus* in the vagina. *Lactobacillus* has been reported to enhance vaginal immunity to pathogens (Greifová et al., 2017). Therefore, the proliferation of *Lactobacillus* can not only maintain the vaginal low pH environment, conducive to the growth of beneficial bacteria. It also enhances the immune function of the vagina. We also observed elevated relative abundance of *Bacteroides* and *Acinetobacter* in the Model group. It has been reported that *Bacteroides* are beneficial bacteria in the intestinal tract, but if they escape to other parts of the body outside the intestinal tract, they act as pathogenic bacteria, leading to abscesses and other infections, such as reproductive tract infections(Zafar and Saier, 2021). *Acinetobacter* is a conditionally pathogenic bacterium that easily causes infection when the body’s resistance decreases. *Acinetobact*er is one of the major opportunistic pathogens causing nosocomial infections, which can cause skin infections, genitourinary tract infections and so on (C. M. Lee et al., 2022).

The beneficial effects of *BCFYQ* on host metpabolism may be related to metabolites produced by vaginal microbes, and we found that *BCFYQ* altered 30 metabolites. It is worth mentioning that we screened out 30 differential metabolites. Among them, phosphorylcholine and betaine, as metabolites of biogenic amine precursors, have anti-inflammatory properties (Rowley et al., 2010; G. Zhao et al., 2018). The results of cluster analysis showed that the content of these two metabolites increased after *BCFYQ* treatment compared with the Model group. Through our pathway enrichment analysis, betaine was associated with betaine metabolism, methionine metabolism, glycine and serine metabolism. Studies have shown that betaine directly affects the concentration of homocysteine ​​by promoting the formation of methionine from homocysteine, and attenuates the stress response induced by homocysteine. At the same time, betaine converts homocysteine ​​into methionine, which plays an important role in antioxidant effects (Glier et al., 2014). In addition, betaine exerts an anti-inflammatory effect by inhibiting the NF-κB signaling pathway and reduces the levels of pro-inflammatory factors such as IL-1β (Veskovic et al., 2019). This is consistent with our cytokine assay results. Arachidonic acid (AA) can be metabolized to prostaglandins (PGs) by the cyclooxygenase pathway. Among them, PGE_2_ has the effect of inducing inflammation, fever and pain. Our results showed that AA was significantly aggregated in the Model group, while its levels were significantly reduced after *BCFYQ* treatment. Interestingly, IL-1 can stimulate phospholipase A2 gene expression and protein synthesis in different cells, and increase its activity, thereby decomposing membrane phospholipids to produce AA, resulting in the generation and release of PGE_2_ (Schalkwijk et al., 1992; Pfeilschifter et al., 1993). It can be seen that the relationship between inflammation and metabolites is not a single relationship, but plays a complementary role.

Our results showed that the concentration of phosphatidylcholine in the *BCFYQ* group was significantly higher than that in the Model group, and pathway enrichment analysis showed that phosphatidylcholine biosynthesis had a significant effect on this pathway. Phosphatidylcholine, the major phospholipid component of eukaryotic cell membranes, is generally known for its anti-inflammatory properties (Treede et al., 2007). And there is increasing evidence that choline metabolites derived from their synthesis and catabolism contribute to proliferative growth and programmed cell death (Ridgway, 2013). In addition, we also observed some metabolite changes related to amino acid metabolism, such as UDP-N-acetylglucosamine and N-Acetylaspartic acid. But overall, *BCFYQ* treatment of mixed vaginitis was more related to lipid metabolism.

We found that *Dethiobiotin* was positively correlated with *Lactobacillus* and negatively correlated with *Escherichia-Shigella* in Spearman’s rank correlation analysis. *Dethiobiotin* is a sulfur-containing water-soluble vitamin that acts as a cofactor in various key cellular metabolic reactions. It has been reported in the literature that *Dethiobiotin* can maintain the integrity and soundness of epithelial tissue structure and has a protective effect on epithelial mucosal tissue. In addition, it is necessary for the reproduction of yeast, *Lactobocillus* and other bacteria. Studies have shown that some chronic inflammatory diseases are associated with biotin deficiency, and biotin deficiency can enhance the inflammatory response of CD4+ T cells (Rodriguez-Melendez et al., 2003; Elahi et al., 2018). Therefore, we preliminarily concluded that *BCFYQ* increased the content of *Dethiobiotin* in vaginal epithelium, which provided better conditions for the growth and reproduction of *Lactobacillus*. At the same time, *Dethiobiotin* can also repair the damaged vaginal epithelial mucosa caused by mixed bacterial infection, and enhance the local immunity and resistance.

Lactic acid, the major end product of *Lactobacillus*-mediated fermentation (O'Hanlon et al., 2013), is a key metabolite for maintaining low vaginal pH and homeostasis in the vaginal microenvironment. Moreover, it has antibacterial and immunomodulatory activities (Aldunate et al., 2015). The results of our vaginal flora showed that *BCFYQ* significantly increased the abundance of *Lactobacillus*. It is worth noting that the relative abundance of *Streptococcus* is higher in the BCFYQ group than in the Model group. *Streptococcus* belongs to the order *Lactobacillus*, which can produce lactic acid by fermenting glucose (Köhler, 2007). However, the lack of experimental results is that lactate was not found in the differential metabolites we screened, which may be a problem of sample size. However, it is undeniable that *Lactobacillus* and its metabolites are one of the key mediators of *BCFYQ* in the treatment of mixed vaginitis.Mixed biofilms formed by bacteria and fungi reduce susceptibility to antimicrobial agents and increase the spread of antimicrobial resistance (Muzny and Schwebke, 2015). Studies have shown that metabolites of boric acid and *Lactobacillus* contained in *BCFYQ* have inhibitory effects on biofilm formation (Ventolini, 2015). We speculate that this is also a key point of *BCFYQ* in the treatment of mixed vaginitis, which is worthy of our further study.

Meng Li et al. investigated the effect of Fufang FuRong Effervescent Suppository on vaginal microecological composition and dynamics in mixed vaginitis(M. Li et al., 2022). Similar to *BCFYQ*, both products are vaginal suppositories derived from traditional Chinese medicine and both contain *Sophora flavescens* and *Stemona japonica*. Although there is a large difference in the species of bacteria at the genus level, this may be due to the ethnic differences in the vaginal flora between humans and rats. But their results show that the FuRong group can significantly increase the abundance of *Lactobacillus*, which is consistent with our results. The findings of the current study have several limitations. First of all, *BCFYQ* is a traditional Chinese medicine compound, the drug composition is complex, it is difficult to determine the main antibacterial drugs. The active components of *BCFYQ* still need to be elucidated. Secondly, the recurrence rate of mixed vaginitis is high (Parsapour et al., 2017), and single drug treatment is difficult to ensure no recurrence of the disease at present. Some studies have conducted intervention experiments on vaginitis recurrence (De Seta et al., 2014), which has a great enlightenment for us. In conclusion, subsequent thorough investigation of downstream mechanisms is essential for future research.

## Conclusions

**Figure 7:**
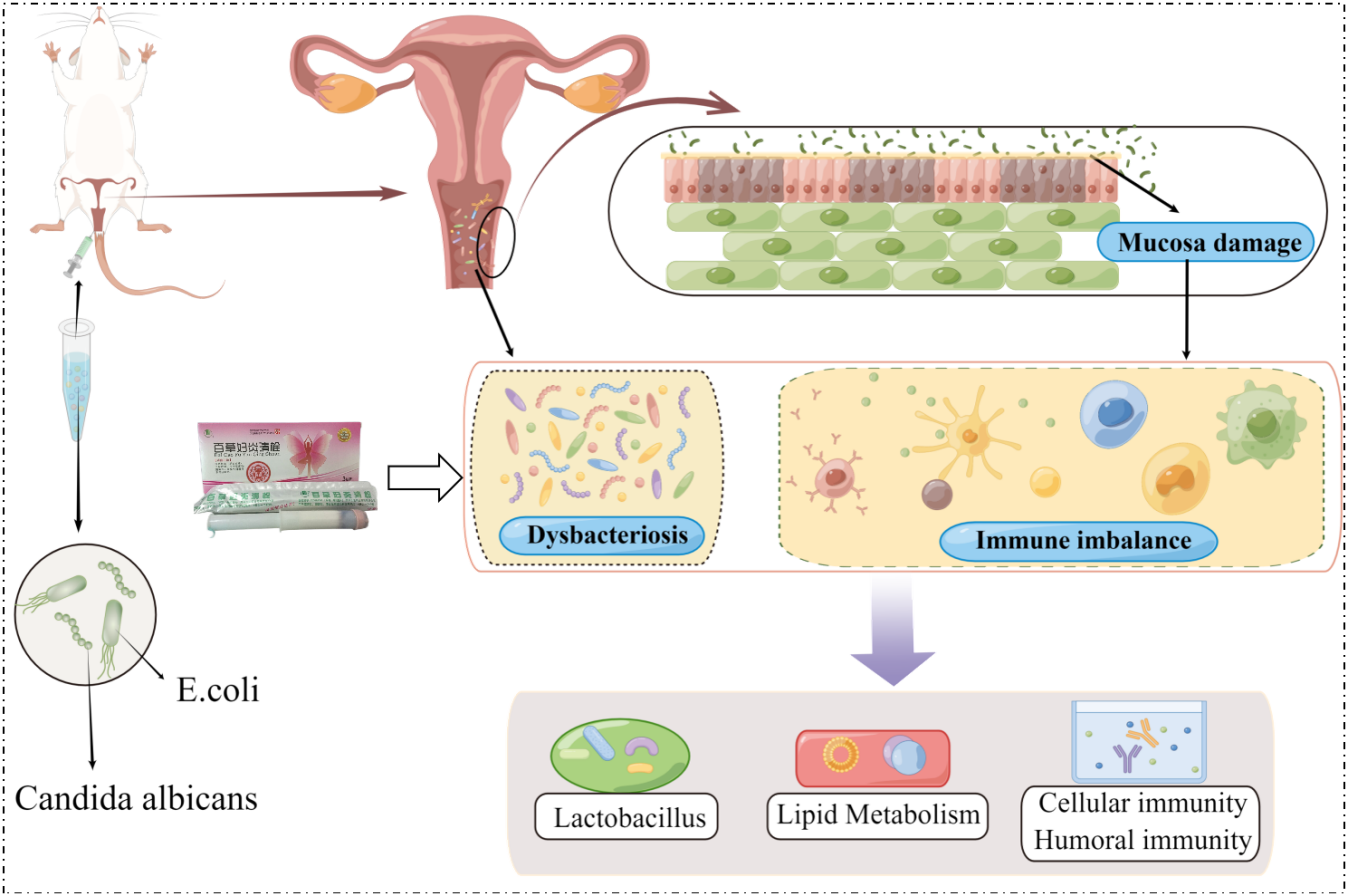
Mechanism diagram of BCFYQ in the treatment of mixed vaginitis.

*BCFYQ* is a multi-component traditional Chinese medicine preparation, which has significant therapeutic effect on mixed vaginitis. *BCFYQ* can improve vaginal flora, inhibit the growth of harmful bacteria, but also protect and promote the growth and reproduction of beneficial bacteria such as *Lactobacillus*. Secondly, *BCFYQ* can repair the damaged vaginal epithelial tissue by affecting the lipid metabolism pathway. This effect can improve the cellular and humoral immune function and reduce the inflammatory response. It is suggested that *BCFYQ* has a significant advantage in the treatment of mixed vaginitis (**Figure 7**). This study explored the mechanism of *BCFYQ* in the treatment of mixed vaginitis, which provided a basis for our further research.

## Data availability statement

The datasets presented in this study can be found in online repositories. The names of the repository/repositories and accession number(s) can be found below: https://www.ncbi.nlm.nih.gov/, PRJNA1009968.

## Ethics statement

The animal study was approved by the Animal Experiment Center of Guizhou Medical University and the Animal Ethics Committee of Guizhou Medical University. The study was conducted in accordance with the local legislation and institutional requirements.

## Author contributions

Participated in research design: QW, PW and XG. Conducted experiments: QW, PW, MY, MZ, SZ, XS, LS, YL, YZ, NJ and XG. Performed data analysis: QW, PW and XG. Wrote or contributed to the writing of the manuscript: QW and PW. All authors contributed to the article and approved the submitted version.
